# What is the rate of incisional hernia? Analysis of initial 100 single-port robotic colorectal procedures

**DOI:** 10.1007/s00464-026-13108-6

**Published:** 2026-08-20

**Authors:** Avihai Moscovici, Tyler Bogaczyk, Jordan Booty, Hien Anh Ton, John H. Marks

**Affiliations:** Division of Colon and Rectal Surgery, Department of Surgery, Lankenau Medical Center, 100 East Lancaster Avenue, Wynnewood, PA 19096 USA

**Keywords:** Single-port robot, Incisional hernia, Colectomy, MIS

## Abstract

**Background:**

As single-port robotic (SP r) colorectal surgery emerges, questions regarding incisional hernia (IH) rates exist. We evaluated our first 100 consecutive SP r colorectal resections to determine postoperative hernia rates.

**Methods:**

From a prospectively maintained database of SP r colorectal procedures, we identified the first 100 consecutive abdominal colorectal procedures. Imaging review was conducted by a blinded radiologist. Clinical examinations were performed by the operating surgeon to detect hernias. Diagnosis of IHs was analyzed as an overall group, by subset with or without postoperative imaging, and by incision position.

**Results:**

Between October 2018 and September 2024, 100 consecutive patients underwent SP r colorectal surgery via an abdominal approach. Mean age was 60.2 years (range 22–84) with a mean BMI of 27.4 kg/m^2^ (range 16.9–42.6). 66% were women. Left-sided procedures were 75% of cases, whereas right-sided procedures were 25%. Mean maximal incision size was 4.84 cm (2.5–8). IH rates by site were as follows: right lower quadrant muscle-splitting incision (*n* = 58), 0%; left lower quadrant muscle-splitting incision (*n* = 16), 0%; umbilical incision (*n* = 10), 20%; stoma site (*n* = 14), 14.3%; and Pfannenstiel incision (*n* = 2), 0%. Postoperative imaging was available for 40% of patients. Mean follow-up was 15.27 months. The overall hernia rate was 4%.

**Discussion:**

In MIS abdominal colorectal surgery, the maximal incision size for colorectal surgery is dictated by the specimen diameter, not the port width. SP robotic surgery did not result in increased IH formation, with a hernia rate of only 4%. All hernias occurred at an umbilical incision site or were stoma associated. No hernias developed in the 76% of cases that a muscle-splitting incision or Pfannenstiel incision was employed. Umbilical incisions should be avoided whenever possible. Concerns regarding increased IH rates for SP r colorectal procedures are unfounded.

Incisional hernia (IH) represents a common long-term complication after colorectal resection, typically developing within the first 12 months following the index procedure. IHs have been shown to have a negative correlation with patient quality of life and results in high expenditure costs for health systems [1, 2]. After open colorectal surgery, more than 20% of patients undergoing midline laparotomy develop an IH [3].

The introduction of minimally invasive techniques into colorectal surgery has been credited with significant improvements in postoperative outcomes, including decreased pain, reduced rates of surgical-site infections, as well as shorter hospital length of stay [4]. Despite the expectation that laparoscopy would reduce IH rates in colorectal surgery, long-term follow-up from randomized trials has not confirmed this benefit [5, 6]. Midline laparotomy incisions and stoma reversal sites are well recognized as high-risk locations for IH development. Hernias following laparoscopic approaches typically arise at specimen extraction sites rather than at trocar sites, particularly when the extraction site is located in the midline [3, 7]. Surprisingly, it has been demonstrated that there is no statistically significant difference in the incidence of IH requiring surgical repair between open and laparoscopic approaches [8].

As robotic surgery gained broader adoption in the colorectal domain, questions arose regarding the potential for hernia developing at 8-mm robotic trocar sites, which are larger than the typical 5-mm ports used in conventional laparoscopy. While the consensus has been that ≥ 10-mm ports have always required fascial closure, the management of 8-mm ports became a topic of considerable discussion [9]. Despite early apprehension that the increased diameter of the incision might elevate hernia risk, this has not occurred. Evidence suggests that the primary determinant of IH formation is the location of the extraction site. Multiple studies have demonstrated that midline extraction sites are associated with substantially higher hernia rates compared with off-midline, muscle-splitting approaches [3].

With the addition of the single-port robotic (SP r) platform for colorectal surgery, concerns have been raised regarding a potential increase in IH rates given the larger trocar diameter (25-mm) required for this technique. With the recent adoption of SP r surgery, there is currently no dedicated literature systematically evaluating postoperative IH following SP colorectal resections. As an early adopter of SP r colectomy, we analyzed our experience to evaluate the risk of IH after SP r colectomy, to determine if there was an effect on IH.

## Materials and methods

From a prospectively maintained database of SP r colorectal operations performed between October 2018 and September 2024 (IRB: E-22-5257), we identified the first 100 consecutive SP robotic intra-abdominal colorectal resections for benign or malignant disease. Right-sided procedures were defined as right colectomy and extended-right colectomy. Left-sided procedures were left colectomy, low-anterior resection (LAR), Hartman’s reversal, proctectomy, rectopexy, and total proctocolectomy. Patients who underwent SP r transanal-transabdominal proctosigmoidectomy, transanal total mesorectal excision or natural orifice transluminal endoscopic surgery were excluded to ensure a homogeneous cohort of purely transabdominal SP cases.

**Fig. 1:**
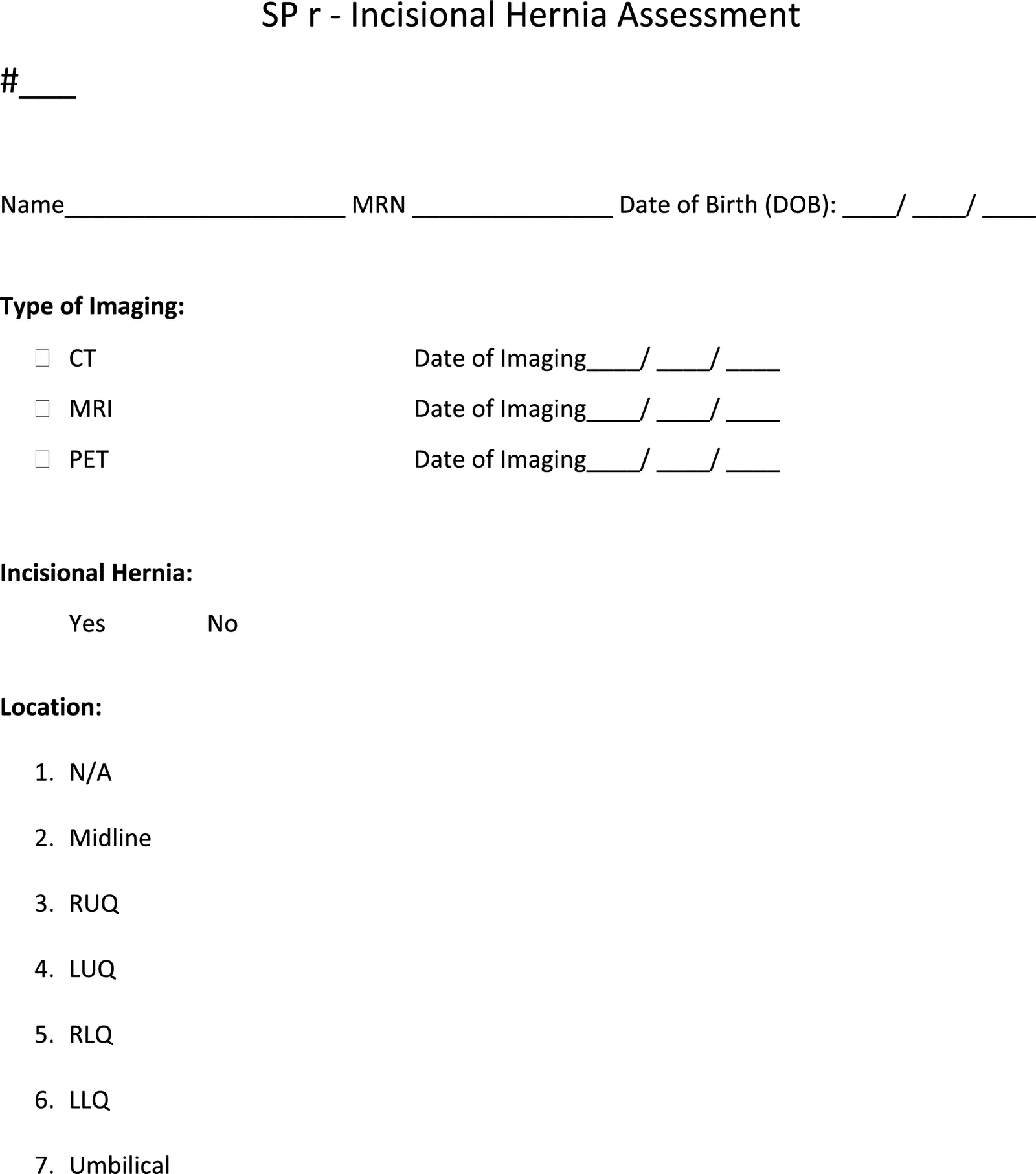
Radiologist assessment form for incisional hernia

Comprehensive data were prospectively collected, including patient demographics, medical history, operative variables, and postoperative outcomes. Data analysis was carried out via Chi square and Fisher’s exact tests with a significance value set at* p* = 0.05. The primary endpoint was the development of an IH. Hernias were identified through clinical evaluation documented in postoperative follow-up notes or, when available, through postoperative image review. Imaging modalities included computed tomography (CT) and magnetic resonance imaging (MRI). All imaging studies were independently reviewed by a radiologist, blinded to patient clinical details, using a dedicated IH assessment form (Fig. 1). Incidental findings of inguinal hernias or previously known hernias were recorded but not assigned as an IH resulting from SP r colectomy.

### Surgical closure technique

The abdominal wall incisions were closed in a standardized, layered fashion. First, the posterior fascial layer was closed using a running 1-PDS suture, followed by thorough saline irrigation of the wound. The anterior fascia was then closed with a running 1-PDS suture. After the wound was irrigated with saline for a second time, the skin was reapproximated using a 4–0 Monocryl subcuticular technique, and Steri-Strips were applied as the final dressing.

## Results

A total of 100 consecutive patients who underwent SP r colorectal resection via an abdominal approach were identified and included in the study. The majority of patients were women (66%), with a mean age of 59 years (range 22–84) and a mean BMI of 26.8 kg/m^2^ (range 16.9–42.6) (see Table 1). 2% of patients were ASA Class I, 65% were ASA Class II, and 33% were ASA Class III. Prior to their SP r operations, 40% of patients had previous abdominal procedures. 75% of the SP r cases were left-sided procedures and 25% were right-sided. The mean maximal incision size was 4.84 cm (2.5–8). Follow-up clinical evaluation was performed in 100% of patients by the attending surgeon, with a mean follow-up time of 15.3 months (1–69.7). There was postoperative imaging available for 40% of these patients, which was reviewed by a blinded radiologist.

**Table 1:** Overall patient demographics

	Mean (range)
Age (years)	59 (22–84)
BMI (kg/m^2^)	26.8 (16.9–42.6)
Maximal incision size (cm)	4.8 (0.6–8.0)
Follow-up (months)	15.3 (1.0–69.7)

	%
Women	66
ASA classification	
Class I	2
Class II	65
Class III	33
Previous abdominal surgery	40

The overall hernia rate was found to be 4%. The SP r incision location and corresponding hernia rates by incision site were as follows: right lower quadrant (RLQ) muscle-splitting incision (*n* = 58), 0%; left lower quadrant (LLQ) muscle-splitting incision (*n* = 16), 0%; umbilical incision (*n* = 10), 20%; stoma site (*n* = 14), 14.3%; and Pfannenstiel incision (*n* = 2), 0% (see Table 2). Of the 4 patients who developed IHs, 50% occurred at an umbilical incision site and 50% were stoma related, as part of the procedure the stoma was reversed and we elected to use this opening to insert the SP robot, or used for port insertion/specimen extraction site to create diverting ileostomy. When comparing the umbilical incision site (*n* = 10) with non-umbilical incision sites (muscle-splitting and Pfannenstiel, *n* = 76), the hernia rates were 20% and 0%, respectively (*p* = 0.01). When comparing stoma sites (*n* = 14) with muscle-splitting and Pfannenstiel incisions (*n* = 76), the respective hernia rates were 14.3% and 0% (*p* = 0.002). There were no hernias that developed at the RLQ, LLQ, or Pfannenstiel incision locations.

**Table 2:** Single-port robotic incision location and hernia rates

Location	Patients *N* (%)	Hernia rate *N* (%)
RLQ muscle-splitting incision	58 (58)	0
LLQ muscle-splitting incision	16 (16)	0
Pfannenstiel incision	2 (2)	0
Umbilical incision	10 (10)	2 (20)
Stoma site	14 (14)	2 (14.3)

Stoma associated hernia fell into two categories. There were 8 patients who had an existing stoma prior to their SP r procedure and 92 patients who did not have a stoma. The hernia rate for patients with existing stomas was 12.5% vs. 3.2% in patients without stomas (*p* = 0.2). Additionally, 11 patients had a protective stoma after SP r procedure, while 89 did not have a stoma. Of the 11 who had a diverting stoma, 1 patient (9%) developed a parastomal IH, compared to 3 patients (3.3% without stomas) who developed an IH that never had a diverting stoma (*p* = 0.3). Five patients with existing stomas (62.5%) were diverted to protect their anastomosis and maintained a stoma postoperatively. Overall, there were 14 patients who had a stoma and 86 patients who never had a stoma. There were two stoma-related IH (14.3%) vs. two IH (2.3%) that developed in patients who never had a stoma (*p* = 0.03) (Table 3).

**Table 3:** Characteristics of non-hernia vs. hernia patients

	No hernia (*n* = 96) Mean (range)	Hernia (*n* = 4) Mean (range)	*p*-value
Age (years)	58.6 (22–84)	67.3 (52–74)	0.15
BMI (kg/m^2^)	26.7 (16.9–42.6)	29.4 (21.8–36.1)	0.29
Maximal incision size (cm)	4.84 (0.6–8.0)	4.8 (4.4–5.5)	0.91
Follow-up (months)	14.9 (1.0–69.7)	28.3 (11.7–48.1)	0.12

	*N* (%)	*N* (%)	*p*-value
ASA classification			0.48
Class I	2 (2.1)	0	
Class II	63 (65.6)	2 (50)	
Class III	31 (32.3)	2 (50)	
Prior abdominal surgery	37 (38.5)	3 (75)	0.14

Pathologic diagnoses included diverticular disease (42%), cancer (32%), polyps (11%), rectal prolapse or colonic inertia (11%), and inflammatory bowel disease (4%). The hernia rate was 9.3% in cancer patients, 25% in IBD patients, and 0% for the other groups (*p* = 0.05). The overall infection rate was 4%. One patient (1%) developed a superficial wound infection and three patients (3%) had intra-abdominal abscesses. The hernia rate for patients with infection was 0% compared to 4.3% for patients without infection (*p* = 1.0).

Of the total procedures (*n* = 100), 25% were right-sided and 75% were left-sided. The hernia rate was 8% for right-sided procedures vs. 2.6% for left-sided procedures (*p* = 0.24).

The hernia rates for cancer patients (*n* = 32) and non-cancer patients (*n* = 68) were 9.4% and 1.5%, respectively (*p* = 0.06). In cancer patients, 17 (53.1%) underwent a right-sided procedure and 15 (46.9%) had a left-sided procedure. The hernia rate for right-sided cancer procedures was 11.7% vs. 6.7% for left-sided cancer procedures (*p* = 0.62).

In right-sided cancer procedures, 3 patients (17.6%) had an umbilical extraction site vs. 14 patients (82.4%) with a non-umbilical extraction site. The hernia rate was 66.7% at the umbilical extraction site vs. 0% at a non-umbilical extraction site (*p* = 0.02).

In left-sided cancer procedures, 5 patients (33.3%) had a diverting stoma, whereas 10 patients (66.7%) did not have diverting stomas. The hernia rate was 20% at the diverting stoma site vs. 0% at non-stoma sites (*p* = 0.33).

In patients with malignancy, 18.8% received neoadjuvant therapy, whereas 81.2% did not receive neoadjuvant therapy. The hernia rate for malignancy patients with neoadjuvant therapy was 16.7% vs. 7.7% in patients without neoadjuvant therapy (*p* = 0.50). 21 (65.6%) of the patients with malignancy had colon cancer and 2 (9.5%) developed an IH. Neither of these 2 patients received neoadjuvant therapy, adjuvant therapy, or had a wound infection. 11 (34.4%) of the patients with malignancy had rectal cancer and 1 (9.1%) developed an IH. This patient underwent neoadjuvant chemoradiotherapy but did not have adjuvant therapy or a wound infection.

One patient had a clinically apparent IH. This patient developed a parastomal hernia after undergoing neoadjuvant chemoradiotherapy and a LAR. As was done routinely, the SP r trocar site was used for specimen extraction and served as the location for the diverting loop ileostomy. The patient did not receive adjuvant treatment and did not develop a wound infection. This hernia was asymptomatic and was radiographically confirmed at 1-month follow-up. The parastomal hernia was repaired at the time of ostomy reversal. The other three hernias were not clinically apparent or symptomatic.

One patient underwent completion proctectomy following a total abdominal colectomy for toxic colitis, which utilized the existing stoma site to gain abdominal access for the SP r. This patient later developed a IH at this site, which was repaired at the time of ostomy reversal.

## Discussion

In the minimally invasive era of colorectal surgery, the risk of postoperative IH is primarily driven by the location of the specimen extraction site. This concept has been consistently demonstrated in the laparoscopic colorectal literature and is further reinforced by the findings of our present study [3, 10]. In SP r surgery, the trocar site also serves as the specimen extraction site and is enlarged to accommodate extraction. While the SP r trocar is more than twice the diameter of the largest conventional laparoscopic or robotic port, it does not translate into a higher risk of IH formation when done off-midline and via muscle-splitting incisions. This apparent paradox is explained by the fact that the extraction site is at the SP trocar incision and is an extension of the 25-mm diameter of the SP trocar. Given that the average colonic diameter is approximately 40 mm, the extraction site necessary to remove the specimen dictates the largest fascial defect, rendering the size of the SP port as a contributor to an IH effectively irrelevant.

Prior meta-analyses of IH following laparoscopic colorectal resections have demonstrated that late abdominal wall closure failures occur predominantly at the extraction site, with midline incisions carrying the greatest risk [3, 7]. Our outcomes are consistent with these prior reports. In our cohort, all observed IHs occurred either at the umbilical extraction site or at stoma sites, where the ostomy had been taken down and the SP inserted or where a new diverting ostomy was created. These locations are already known to be predisposed to hernia formation. In contrast, no IHs were identified at muscle-splitting or Pfannenstiel incision sites.

While it is well demonstrated in the literature that infection and neoadjuvant therapy can lead to IHs, we did not find this in our study [11]. There was no statistically significant relationship between infection and hernia development (*p* = 1.0) or neoadjuvant therapy and hernia development (*p* = 0.50). There was also no significant impact on the hernia rate based on cancer diagnosis (*p* = 0.06) All the right-sided hernias occurred at umbilical extraction sites and all the left-sided hernias were stoma related. This highlights the point that extraction site location is the most important risk factor for IH development. During our initial year of experience with the SP r platform, right colectomies were performed using an umbilical incision for both docking and specimen extraction, predominantly for cosmetic considerations. However, the development of paraumbilical IH in the first and fourth patients who underwent this approach prompted reevaluation of our technique. After 12 months, we shifted our practice to primarily muscle-splitting incisions, after which no IH was observed in the subsequent cases. We decided that cosmesis was not worth an increased risk of hernia development and now recommend that umbilical hernia incisions should be used sparingly.

The present study adds meaningful data to a field that remains largely uncharted. With a rapid adoption of SP r technology, literature evaluating IH specifically after SP colorectal resections does not exist. To date, only one publication has compared IH risk between multi-port and SP robotic platforms, and that study was performed by urologists in the context of transperitoneal radical prostatectomy. While this is of interest in the discussion of SP r hernia rates, there is a fundamental difference between SP r colectomy and prostatectomy with respect to hernia formation, largely attributable to the smaller specimen size in prostatectomy. No statistically significant difference in overall IH rates was noted between the groups (SP 8.1% vs. multi-port 9.2%) in the prostatectomy study [12].

When performing a colorectal procedure with the SP r on a patient with either an existing stoma or the need for a protecting stoma, it is advisable to use the stoma site as the docking site to avoid creating additional incisions. Stoma sites are known to have an increased risk for hernia formation and we believe this is unrelated to whether the stoma is used for SP r docking [7]. Therefore, creating an additional incision at another site for SP r docking is not advisable. Regardless, our overall hernia rate was 4%, which is well within reported IH rates in the literature of 2–16% for MIS colorectal procedures [13]. This leads us to conclude that SP r colorectal procedures do not contribute to increased hernia rates.

Our study has limitations. Primarily, all procedures were performed by a single high-volume SP colorectal surgeon, which has the potential to limit generalizability. SP r colectomy is new and enables potential variability in surgical technique and outcomes across institutions and surgeons with differing levels of experience. Additionally, not all patients underwent routine postoperative imaging, which could underestimate radiographically occult hernias. Nonetheless, our methodology aligns with contemporary IH research, where clinically relevant hernias remain the primary endpoint.

## Conclusions

This study provides early and clinically significant evidence that SP r colorectal surgery is associated with a low incidence of postoperative IHs. The trocar should be placed off-midline, via a muscle-splitting incision, and should serve as the extraction site. Our findings reinforce that the extraction site, rather than trocar size, is the primary determinant of IH risk in minimally invasive colorectal surgery. Additionally, although stoma sites may carry higher IH risk, they should still be utilized for the SP trocar and specimen extraction, since this abdominal wall defect is preexisting and its closure is required regardless.

We hope these findings will help inform future best practices for operative approaches in SP r colorectal surgery. As the SP r platform continues to gain traction in colorectal surgery, findings from this study should be confirmed in larger, multicenter trials with longer postoperative follow-up to establish robust, evidence-based guidelines for SP r colorectal surgery. As it stands, in this report of one of the largest SP r colectomy series in the literature to date, the findings suggest that concerns regarding increased IH rates, due to the 25-mm trocar for the SP robot, are unfounded in SP r colorectal procedures.
